# Univariate Analysis of Short-Chain Fatty Acids Related to Sudden Infant Death Syndrome

**DOI:** 10.3390/diagnostics10110896

**Published:** 2020-11-02

**Authors:** Carlos E. Galván-Tejada, Karen E. Villagrana-Bañuelos, Laura A. Zanella-Calzada, Arturo Moreno-Báez, Huizilopoztli Luna-García, Jose M. Celaya-Padilla, Jorge I. Galván-Tejada, Hamurabi Gamboa-Rosales

**Affiliations:** 1 Unidad Académica de Ingeniería Eléctrica, Universidad Autónoma de Zacatecas, Jardín Juarez 147, Centro, Zacatecas 98000, Mexico; ericgalvan@uaz.edu.mx (C.E.G.-T.); kvillagrana@uaz.edu.mx (K.E.V.-B.); morenob20@uaz.edu.mx (A.M.-B.); hlugar@uaz.edu.mx (H.L.-G.); jose.celaya@uaz.edu.mx (J.M.C.-P.); gatejo@uaz.edu.mx (J.I.G.-T.); 2 LORIA (INRIA, CNRS), Campus Scientifique BP 239, Université de Lorraine, 54506 Nancy, France; laura.zanella-calzada@univ-lorraine.fr

**Keywords:** sudden infant death syndrome, short-chain fatty acids, univariate analysis, generalized linear model

## Abstract

Sudden infant death syndrome (SIDS) is defined as the death of a child under one year of age, during sleep, without apparent cause, after exhaustive investigation, so it is a diagnosis of exclusion. SIDS is the principal cause of death in industrialized countries. Inborn errors of metabolism (IEM) have been related to SIDS. These errors are a group of conditions characterized by the accumulation of toxic substances usually produced by an enzyme defect and there are thousands of them and included are the disorders of the β-oxidation cycle, similarly to what can affect the metabolism of different types of fatty acid chain (within these, short chain fatty acids (SCFAs)). In this work, an analysis of postmortem SCFAs profiles of children who died due to SIDS is proposed. Initially, a set of features containing SCFAs information, obtained from the NIH Common Fund’s National Metabolomics Data Repository (NMDR) is submitted to an univariate analysis, developing a model based on the relationship between each feature and the binary output (death due to SIDS or not), obtaining 11 univariate models. Then, each model is validated, calculating their receiver operating characteristic curve (ROC curve) and area under the ROC curve (AUC) value. For those features whose models presented an AUC value higher than 0.650, a new multivariate model is constructed, in order to validate its behavior in comparison to the univariate models. In addition, a comparison between this multivariate model and a model developed based on the whole set of features is finally performed. From the results, it can be observed that each SCFA which comprises of the SFCAs profile, has a relationship with SIDS and could help in risk identification.

## 1. Introduction

Sudden infant death syndrome (SIDS) is defined as the death of a child under one year of age, during sleep, without apparent cause. Several investigations have been carried out and the concept of SIDS has changed in order to discover the specific reason that causes death, thus it can be defined as the sudden death of an infant under one year of age, while asleep, which remains unexplained after a thorough investigation, including performance of a complete autopsy, examination of the death scene, and review of clinical history [1,2,3,4].

According to the Centers for Disease Control and Prevention (CDC) in the United States there are 3600 sudden unexpected infant deaths [5]. In Mexico there are around 350 child deaths per year, corresponding to approximately one out of every two thousand born, during the first year of life [6]. Unfortunately in Mexico, as in other countries, the statistics on the frequency of SIDS are not real, either due to under-registration or misdiagnosis [7].

SIDS is the main cause of death in previously healthy babies [8], as of now there is no consensus on the reasons that causes it [9] which results in uncertainty when not being able to identify which babies are at risk of suffering it, and therefore of suffering a lethal episode, also known as episodes of apparent life threatening events (ALTE, “an episode that is frightening to the observer and that is characterized by some combination of apnea (central or occasionally obstructive), color change (usually cyanotic or pallid but occasionally erythematous or plethoric”) [10].

There are data on SIDS, but they provide no explanation for the etiology and pathogenesis of sudden infant death. Sudden infant death is considered “multifactorial” [11] and this concept, however, helps as little as the term “idiopathic”, which is used in many other diseases to explain that something does not have an explication yet. As our knowledge of SIDS grows, it will be possible to recognize and classify pathological conditions behind the sudden death of a child [12].

Several investigations have been carried out from all possible areas, such as engineering [13,14], epidemiology [15], pathophysiology [16], pathology [2], pediatrics [17], neurology [18], among others in the search for solutions to it. Among the associated conditions, inborn errors of metabolism (IEM) stand out [19,20].

IEM are a group of conditions characterized by the accumulation of toxic substances usually produced by an enzyme defect [21]. It can also be defined as a diverse heterogeneous group of disorders with protean clinical manifestations presented mainly in the pediatric population [22]. Disorders of the β-oxidation cycle are included in the group of diseases that include IEM: Acetyl CoA is generated from fatty acids through repeated cycles of β-oxidation. Groups of four specific enzymes are required for different chain lengths (very long chain, long chain, medium chain, and short chain) [21]. Thus one of the pathologies that could explain SIDS are the enzymatic defects to metabolize short chain fatty acids (SCFAs), these being comprised of 1–6 carbon based anions, which are produced during bacterial fermentation, of which, acetate (C2), propionate (C3), and butyrate (C4) are the most abundantly produced. SCFAs are produced naturally within the colon by fermentation of carbohydrates, both dietary and endogenous, and proteins that are accessible to the microbiota [23].

There are some investigations that have related the IEM with SIDS or ALTE [20,24], Works such as those of Villoria et al. [25], mention that short-chain fatty acid problems are considered among the possible causes of this syndrome, however the statistics between the relationship are not established due to methodological problems.

IEM are currently considered treatable pathologies if they are diagnosed promptly [26]. In the pediatric age from the first hours of life they can show symptoms and signs similar to other diseases, or even have no symptoms. which makes them difficult to diagnose, this leads to sequelae such as malnutrition, seizures, mental retardation, apnea [24], and ALTE, which could also compromise consciousness (lethargy and drowsiness, which leads to deep coma and death) [24].

The incidence of IEM as a cause of SIDS or ALTE remains unknown (or, at least, not well known) [24], however if proper investigations are not carried out, these cases often meet the criteria for SIDS [27]. There are controversies regarding the proportion of SIDS attributable to fatty acid oxidation defects. It is mentioned that there are no reliable records of SIDS, the investigations could have biased data such as differences in the selection of cases, and the type of methodology, among others [27]. As previously mentioned, when diagnosed these disorders can be treated in a timely manner.

SIDS, as mentioned before, is a diagnosis of exclusion, although it means that the reasons why this occurs have not been discovered yet. With the advent of advances in science, opportunities are opened to observe, analyze, and find relationships among the possible risk factors.

A proposal that has increased the analysis of relationships between variables and SIDS is the application of artificial intelligence techniques, such as machine learning (ML), looking for the development of computed-aided diagnosis (CAD) tools, and seeking to counter the points mentioned above. Kononenko, I. [28] mentioned how artificial intelligence, which is part of computing, tries to make computers smarter based on learning. Most of the current research agrees that it is not smart but that it learns. That is why to create this type of learning model, based on the capacity of what was previously learned, it is necessary that the data provided to the computer be reliable, as complete as possible for an adequate characterization of the disease and so to predict and diagnose.

In this sense, CADs have demonstrated to be useful in many fields of human medicine to aid in diagnostics [29,30,31,32], but it is important to highlight that their role in pediatrics; Kokol et al. [33] has detected the relevance that artificial intelligence has had in the pediatric area, since 2013 and beyond that has focused on the application of ML in diseases like schizophrenia, pneumonia, asthma, abnormality, and epilepsy. No new ML approaches emerged, however the assessment of accuracy with the introduction of new metrics has become important and the research focus has shifted from classification to predictive models. For example, Mitchell et al. [33] developed a predictive model in children with acute lymphoblastic leukemia. It consisted of a predictive model for identifying the increased risk for thromboembolism. It would be beneficial in targeting interventional studies to high-risk groups. It was evaluated in 456 children and then validated in 339 patients and the model’s specificity was 96.2% and its sensitivity was 63.2%, on the basis of high specificity, the model may identify children with leukemia at risk of thromboembolism. Mueller et al. [34] used a dataset of 486 mechanically ventilated premature infants to develop predictive models and determine whether machine learning methods can predict the extubation outcome in premature infants as well as clinicians using machine learning algorithms such as artificial neural networks (ANN), support vector machine (SVM), naive Bayesian classifier (NBC), powered decision trees (BDT), and multivariate logistic regression (MLR). Finding that for some models (ANN, MLR, and NBC) in the IEM study, the ML techniques also had satisfactory results (area under the receiver operating characteristic curve (AUC): 0.63–0.76). There are works that use classification algorithm methodologies with random forest (RF) and include metabolic, socioeconomic, demographic characteristics, among others, to help with the prognosis, such as the work of Wandhwani et al. [35] who applied the above to know the prognosis in the liver transplants of pediatric patients.

Therefore, in this work the analysis of postmortem SCFA values of babies with and without SIDS, based on the development of univariate models through a generalized linear models approach (GLM), is proposed as the main objective. Based on an univariate statistical analysis and a multivariate comparison, the intention is to contribute to a future assisted diagnosis to support in the diagnosis of the risk of suffering ALTE without reaching SIDS.

This paper is organized as follows. Section 2 presents a detailed description of the SCFA profile of infants data set used, as well as the methods applied for the univariated analysis to find relationships. Section 3 presents the experiments performed using the eight short fatty acids as well as the results of the logistic regression and the evaluation of each with the receiver operating characteristic curve (ROC curve) and AUC. In Section 4, a discussion about the results of the relation of SFCA and SIDS is described and presents the conclusions of the work.

## 2. Materials and Methods

In this study, a public database available from the NIH Common Fund’s National Metabolomics Data Repository was used. This study is summarized as an *Analysis of SCFA profile in infants dying of SIDS compared to infants dying of controls* from the University of Michigan, Biomedical Research Core Facilities [36]. The criteria to select this dataset were:

(1) Case-control study;
(2) Short-chain fatty acids profile for cases and controls;
(3) SIDS related study.

Given these constraints, only one public dataset was found. This dataset is described in detail below.

### 2.1. Data Description

The dataset denominated “SCFA profile in babies dying from SIDS” [36], containing information referred to 18 patients, 5 controls, and 13 patients diagnosed with SIDS. This information includes the values obtained for the features presented in Table 1 which were calculated postmortem through cold extraction measured by electron ionization-gas chromatography mass spectrometry (EI GC-MS) without derivatization. The unit of these values is uM. For more details on the process that was used to prepare the sample, the description in the original project can be consulted [36].

### 2.2. Data Availability

This data is available at the NIH Common Fund’s National Metabolomics Data Repository (NMDR) website, the Metabolomics Workbench, https://www.metabolomicsworkbench.org where it has been assigned Project ID PR000512. The data can be accessed directly via the Project DOI: 10.21228/M8GQ27. This work is supported by NIH grant U2C-DK119886 [36].

### 2.3. Data Preprocessing

Data pre-processing consisted of forming a tidy data, which included each of the short chain fatty acids and the postmortem interval. In addition, the gestational age and postnatal age information was analyzed, identifying that these data did not agree with reality, since the gestational age at birth ideally covers a maximum of 42 weeks of gestation, so that when these weeks are exceeded, it puts the life of the binomial mother-son at risk [37]. Therefore, a possible error was identified in the labeling of said data, so the original data for said ages, provided by NIH Common Fund, were inverted, changing the values referring to the gestational age for those referring to postnatal age and the opposite.

### 2.4. Generalized Linear Model

A GLM represents a generalization of a linear model through expanding its scope to nonlinear data that can be transformed into a linear form using suitable transformations. GLMs allow one to handle the limitation that linear regression presents, which is the assumption of linear relationships between the input and output. The nonlinear relationship between the input and output is converted into linear by adding a step of transforming part of the data (the input or the output) into another domain. This step is known as the basis function. One of the basis functions that is widely used is logistic regression, which uses a logistic function to transform the nonlinearity into linear. In the logistic function, the output is also mapped between a range of [0, 1], being equivalent to a probability density function.

In logistic regression, an exponential functional to the linear regression output is added, $y_{i}\in R$, to constrain it into $y_{i}\in$ [0, 1]. The relationship between the input and predicted output can be calculated with Equation (1).

(1) $$\hat{y_{i}}=\sigma \left(\sum_{j=1}^{n}x_{i,j}.w_{j}+w_{0}\right).$$

As the output obtained by logistic regression presents a symmetrical distribution between [−∞,∞], it is better suited for classification problems [38].

### 2.5. Validation

In this study, there are only two possible outputs for the classifier, “positive” and “negative”. These possible outputs can be represented within a confusion matrix, which allows one to observe the differences between the positive and negative predicted by the classifier, as well as the correct class of each case.

This matrix allows an observation of true positives (TP), true negatives (TN), as well as the false positives (FP) and false negatives (FN). Based on this, different metrics can be estimated to help measure the performance of the model under different criteria. Specifically, there are two metrics that can be calculated. Firstly, sensitivity can be calculated by Equation (2):

(2) $$Sensitivity=\frac{TP}{TP+FN},$$

which describes the proportion of true positives, i.e., the probability that a case of SIDS will be correctly classified. And secondly, specificity [39], which can be calculated by Equation (3),

(3) $$Specificity=\frac{TN}{FN+TP},$$

which describes the proportion of true negatives, that is, the probability that a case of non-SIDS will be correctly classified.

Therefore, to evaluate the results of the proposed analysis, an approach was carried out using the ROC curve. The ROC curve is a well known technique based on the metrics mentioned before [40], used to visualize the performance of a classifier, complemented further by the area under the ROC curve (AUC), which is dependent on a decision point (threshold). The AUC value represents the probability that a random positive sample is correctly identified and it represents a desirable measure since it is scale-invariant (measuring the ranking of the predictions instead of their absolute values) and its classification-threshold invariant (measuring the prediction’s quality irrespective of the threshold selected).

## 3. Experiments and Results

This section presents the experiments performed for the development of this work as well as the results obtained.

Initially, the set of features contained in the dataset were submitted to an univariate analysis, developing a model based on the relationship between each feature and the outcome, obtaining 11 univariate models. Then, each model was validated calculating their ROC curve and AUC value. For those features whose models presented an AUC value higher than 0.650, a new multivariate model was constructed, in order to validate its behavior in comparison to the univariate models. In addition, a comparison between this multivariate model and a model developed based on the whole set of features is finally performed.

Table 2 presents the 11 features used for this work together with the AUC values they obtained through the univariate analysis performed, based on a GLM approach. Features are listed in ascending order according to the AUC value calculated for each.

**Figure 1:**
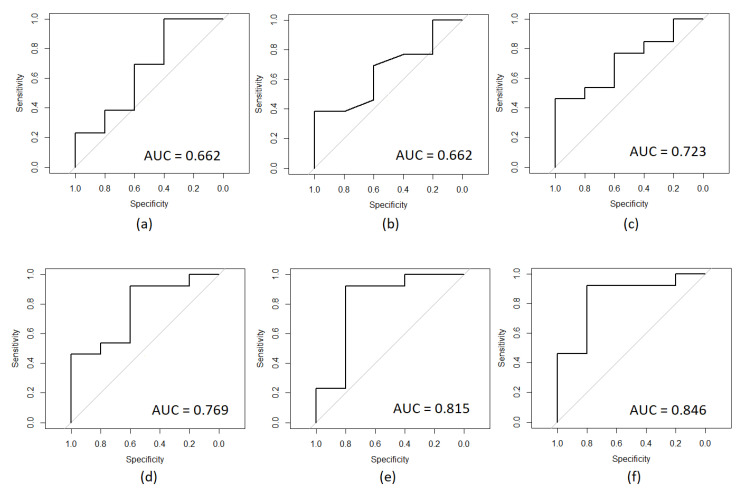
ROC curves obtained through the univariate models of the features that presented an AUC value higher than 0.650: (**a**) Isubutyric acid, (**b**) postnatal age weeks (**c**) butyric acid, (**d**) hexanoic acid, (**e**) valeric acid, and (**f**) acetic acid.

Figure 1 presents the ROC curves corresponding to the univariate models that reached an AUC value higher than 0.650. In (a) it is shown that the ROC curve belonging to the model developed for the isubutyric acid feature, in (b) for the postnatal age weeks feature, in (c) for the butyric acid feature, in (d) for hexanoic acid, in (e) for valeric acid feature and, and in (F) for acetic acid.

**Figure 2:**
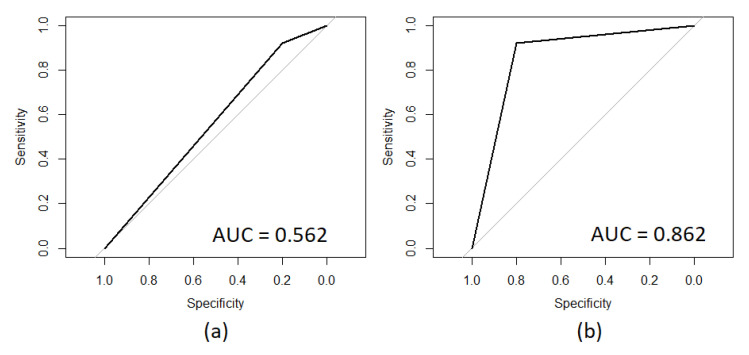
ROC curves obtained through (**a**) the model containing the complete set of features and (**b**) the model containing the set of features presenting an AUC value higher than 0.650.

Figure 2 presents the ROC curve obtained for the model based on the total set of features in (a), while (b) presents the ROC curve obtained for the model based on the set of features that presented an AUC value higher than 0.650.

From the results, it can be observed that each SCFA which comprise the SCFAs profile has a relationship with SIDS, according to the validation metric used, AUC, presented in Table 2. However, only the models of those features that presented an AUC value higher than 0.650 were taken into account for a subsequent multivariate evaluation, since the probability that they correctly identify a positive patient is higher than 0.65%, being statistically significant. For these features, the ROC curve is shown in Figure 1, where it can be observed that not only did the series of fatty acids, isobutyric, butyric, hexanoic, valeric, and acetic, present a significant value for the identification of positive patients, but also for the postnatal age in weeks. Regarding these SCFAs, the valeric acid and acetic acid were those that obtained higher AUC values, presenting 0.815 and 0.846, respectively, showing that based on the information contained in these features, the probability that a positive patient was correctly identified is higher than 81%. On the other hand, the isobutyric acid was the one that obtained a lower AUC, presenting a value of 0.662, meaning a patient who was identified as positive has a 60% probability of having a correct classification. It is remarkable that the feature containing information about the postnatal age obtained an AUC value equal to the one obtained by the feature of the isobutyric acid, meaning that both are presenting the same contribution in the correct identification of positive patients, according to this value however, according to the ROC curves, it can be seen that the behavior of these is evidently different, which represents that the contribution of both features is not applied uniformly. That is to say that while the model with the isobutyric acid might be correctly identifying a set of patients, the model of the postnatal age in weeks might be correctly identifying a different set.

The AUC descriptor was selected because it is presented in the bioinformatics and biomedical literature [41,42,43,44,45] as a tool to describe the behavior of the phenomenon, giving a metric that, if it is greater than 0.5 it is considered useful for describing the phenomenon, in this case the relationship of short-chain fatty acids and SIDS.

Therefore, based on the results it is notable that the behavior of a model developed from the full set of features does not generate any contribution for the identification of positives. However, this does not mean that the features by themselves do not contain information that is representative for the identification of positives, but that modeled together they do not achieve significant generalization. Therefore, if an analysis is carried out univariately to identify the contribution of each feature, in order to know which ones are representative for the correct identification of positives, as was done in this work, it is possible to rule out those that do not generate a contribution and keep only those that do. In this way, the number of features necessary for the identification of the target is reduced, eliminating unnecessary, redundant, or non-contributing information, and a generalized model is developed, allowing one to identify SIDS with a significant probability of correctness.

## 4. Discussion and Conclusions

In this work, it was identified that a multivariate model, as presented in other bioinformatic and biomedical approaches research [46,47,48], presented better results compared to an univariate model. However, the univariate model was necessary to identify the contribution of each characteristic and conform the multivariate model. It was shown that the characteristics of babies who died from SIDS, using values of short chain fatty acids, were able to classify cases of death for SIDS. Therefore, these results show that, in addition to the SCFAs that better relate to SIDS, the age of the infant in weeks contributes in the identification of positives.

It stands out, that if a patient is labeled as SIDS, after of exhaustive investigation according to the definition in the literature, metabolic diseases were ruled out. The results were interesting, due to low probability rate of metabolic diseases [49], even more, if the subject was subjected to medical research, as the criteria to classify it as death from SIDS mark it [50]. Nevertheless, there are discrepancies between countries regarding the protocols for investigating cases of death from SIDS [51].

The relationship between IEM and potential death from SIDS is well known and has been described in the literature for three decades [25]. However, it is important to point out the high discrepancies between countries for neonatal screening of metabolic diseases and as there are thousands of them [25], to examine each patient for each metabolic disease. In Europe, national programs, the screening of metabolic diseases vary by region ranging from two up to 30. In addition regions of America, Asia, the Middle East, and North Africa [25], present similar situations. The metabolic disorders that are investigated in most countries include: Phenylketonuria, congenital hypothyroidism, congenital adrenal hyperplasia, cystic fibrosis, galactosemia, biotinidase deficiency, and acyl-CoA deficiency medium chain dehydrogenase [52]. As can be seen, programs in search of diseases related to defects in SCFA metabolism are not routinely included in most countries. In addition, due to the nature of these disorders and the complexity of both the type of substances and chemical steps in a certain metabolic pathway, they are not suspected and are hardly diagnosed [53]. It is relevant that pathologies such as medium chain fatty acid disorders are analyzed in less than fifty percent in regions of Europe and America [25], being one of the main disorders associated with SIDS, which allows one to deduce that a small percentage of research is carried out for short chain disorders. It would be convenient to take these variables into consideration in subsequent studies. Another point to consider is that a part of physiological regulation involves metabolites such as SCFAs, which is accessed through the intestinal epithelium interacting with the cells. These acids not only serve as energy for the intestinal microbiota, but also for the intestinal epithelial cells, having functions of regulators in the physiology and immunity of the host, resulting in beneficial metabolites with anti-inflammatory capacity [54]. Furthermore, breastfeeding could be associated with the concentrations of short chain fatty acids, either by fetal maternal transfer, or by the type of bacterial colonization of the colon, which is widely recognized as an immune benefit of exclusive breastfeeding, which could contribute to the risk of suffering SIDS [55]. Together with this research, this supports that the disorders related to SCFAs should be investigated, since they have a fundamental role in immunity and are able to characterize SIDS through the multivariate model.

Nevertheless, there are several issues that must be considered in this study:

- This case-control study was performed using a dataset from the University of Michigan, in the Boston children’s Hospital. From the literature, we are aware that NBS differ methodologically and in the disorders screened worldwide, meaning that this study can not be generalized onto the worldwide population. Several factors must be considered when the results are generalized, among them the profiles of SCFAs that were obtained to perform the data-set, which were postmortem. Thus, it is unknown if the found values fluctuate as the postmortem interval increases, and therefore whether they differ or not from a living subject. It is also recognized that race could also influence these values, since it has been described in various investigations that the prevalence is higher in certain race groups as opposed to the white race. In addition to racial characteristics, environmental influence, population lifestyles, access to health services (prenatal care, childbirth care, well-child care), socioeconomic status, among many other factors that vary according to the population, the territory must also be considered, which can directly and indirectly affect the appearance of SIDS;
- As described in Section 2, this study is comprised of 18 subjects, which is prone to overfitting even when a blind-test approach is performed to validate experimentation. As this is a small number of cases, valuable features for case identification could be excluded. One of them is that most of the study subjects were close to one year of age, so there could be a risk of overfitting and not identifying younger patients, since a higher range of risk has been identified in the literature, which is between the ages of 2 and 8 months, data that varies according to the authors, this age could mark significant differences in SCFAs levels according to weeks of life;
- As presented in Section 3, the SCFAs profile gives us insights to possible SIDS complications, however, we are aware that SIDS is a complex, multifactorial disorder, which can be influenced by other risk factors, it being a disease described as a syndrome and remembering that the concept refers to a set of signs and symptoms that characterize a disease, so the absence or presence of a particular sign or symptom is not decisive for suffering it. However, since several symptoms or signs are present in a patient, they have become relevant for their study. Until now, the most significant factors that describe this pathology have not been identified. Thus, long lists grouped into genetic or inheritance factors, maternal factors, environmental factors, and newborn factors can be found, just to mention some. It has been shown that each of them could intervene in the presentation of SIDS;
- Therefore, other clinical data from patients is not available in this study and can influence the results. Among which stands out the way of obtaining the product of conception, that is, by delivery or cesarean, which when compared will have different types of bacterial colonization, and remember that the production of SCFAs depends largely on the fermentation of food by part of the bacteria in the colon. This is also influenced by the type of feeding, whether exclusively breastfeeding, supplementary, or combinations of both. In the same sequence of ideas, the age of ablation and weaning have an important role in variations in the gut microbiota among newborns.

Its possible to conclude that the SFCAs profile allows the identification, in a quantitative way, of the risk of SIDS. The risk identification could be done with acetic acid, followed by valeric acid. In addition, a particular SFCAs profile comprised by isobutyric, butyric, hexanoic, valeric, and acetic acids, complemented by the postnatal age in weeks, could improve risk identification. The delimitation of which risk factors are most significant for a possible appearance of SIDS is very relevant, and the foregoing opens opportunities for the identification of risk factors with greater statistical significance and, therefore, useful for creating, in the future, models with greater complexity that involve a greater number of variables, so as to be able to predict with greater certainty the risk of suffering ALTE or SIDS. Research in this sense will become even more relevant as it is useful information for the health sector and society in general, which will make it possible to strengthen prevention and create timely diagnosis strategies that contribute to reducing the mortality rate.

## Figures and Tables

**Table 1:** Features contained in the “short chain fatty acids (SCFAs) profile in babies dying from sudden infant death syndrome (SIDS)” dataset.

	Feature
1	Postmortem interval PMI hours
2	Gestational age weeks
3	Postnatal age weeks
4	Isovaleric acid
5	Octanoic acid
6	Propionic acid
7	Isobutyric acid
8	Butyric acid
9	Hexanoic acid
10	Valeric acid
11	Acetic acid

**Table 2:** AUC values obtained by each feature through the univariate analysis.

Feature	AUC Value
Isovaleric acid	0.508
Octanoic acid	0.538
Gestational age weeks	0.592
Postmortem interval PMI hours	0.600
Propionic acid	0.646
Isobutyric acid	0.662
Postnatal age weeks	0.662
Butyric acid	0.723
Hexanoic acid	0.769
Valeric acid	0.815
Acetic acid	0.846
